# Real-time PCR data for reference candidate gene selection in tomato infected with Tomato curly stunt virus

**DOI:** 10.1016/j.dib.2020.105750

**Published:** 2020-05-21

**Authors:** Mamokete Bokhale, Imanu Mwaba, Farhahna Allie

**Affiliations:** a Department of Biochemistry, University of Johannesburg, Auckland Park, Johannesburg, 2006, South Africa

**Keywords:** Tomato, ToCSV, Real-time PCR, Reference genes, Normalization, Melt curve analysis

## Abstract

Real-time PCR (qPCR) is a useful and robust method of quantifying gene expression, provided that suitable reference genes are used to normalize the data. To date, suitable reference genes have not been validated for tomato gene expression changes in response to Tomato curly stunt virus (ToCSV). RT-qPCR was conducted on resistent (R) and susceptible (S) tomato leave tissue infected with ToCSV at 35 days post infection. Ten candidate reference genes were selected and validated using SYBR green. Here, we report a set of primers designed for the ten candidate genes and the data for the melt curve analysis and standard curves generated for each candidate reference gene. This data provides a useful resourse in reference gene selection for future use in the normalization of qPCR data investigating tomato-virus interactions. To our knowledge, this data provides the first selection and testing of candidate reference genes in a tomato-ToCSV pathosystem.

Specifications table

**Table:** 

**Subject**	Biochemistry, Genetics and Molecular Biology
**Specific subject area**	Molecular Biology
**Type of data**	Table, Graph
**How data were acquired**	Data was acquired using real-time PCR using the Bio-Rad CFX Connect Real-Time System. Melt curve analysis was carried out using the Bio-Rad CFX manager 3.1
**Data format**	Raw , Analysed
**Parameters for data collection**	Real-time data was acquired by following manufacture instructions and guidelines (Bio-Rad).
**Description of data collection**	Ten reference candidate genes were selected based on previous qPCR experiments conducted in tomato [1, 2] (Table 1). These candidate reference genes were tested and amplified using qPCR with SYBR green in tomato leaf tissue infected with ToCSV. Melt curve analysis was performed for each of the genes. A standard curve for each candidate reference gene was also generated in order to calculate the PCR efficiency of each primer set.
**Data source location**	City: Johannesburg, Country: South Africa, Latitude: 26° 11’ 7.1334’’, Longitude: 27° 59’ 50.8374’’
**Data accessibility**	All nucleotide sequences used for Primer design using the IDT PrimerQuest tool (https://eu.idtdna.com/PrimerQuest/Home/Index) can be accessed via Sol Genomics Network (https://solgenomics.net/) using the accession numbers in Table 1. All raw data files aquired for qPCR experiments for each of the genes tested are available via Mendeley data [3]. Repository name: Mendeley Data, Data identification number: **DOI:** 10.17632/ccb5mkpkz5.1, Direct URL to data: [https://data.mendeley.com/datasets/ccb5mkpkz5/1]

## Value of the Data

• This data is extremely useful as it is the first report of reference genes tested and analysed in a tomato-ToCSV pathosystem.
• This data set would be valuable to anyone conducting RT-qPCR experiments in tomato as it serves as a good starting point as to which reference genes may serves as suitable candidate genes.
• This data can be further used to optimize and investigate gene expression changes in tomato infected with a number of related geminviruses.
• The data helps illustrate the importance of testing reference genes in near-isogenic lines (NIL) lines in response to virus infection.

## 1 Data Description

**Fig. 1:**
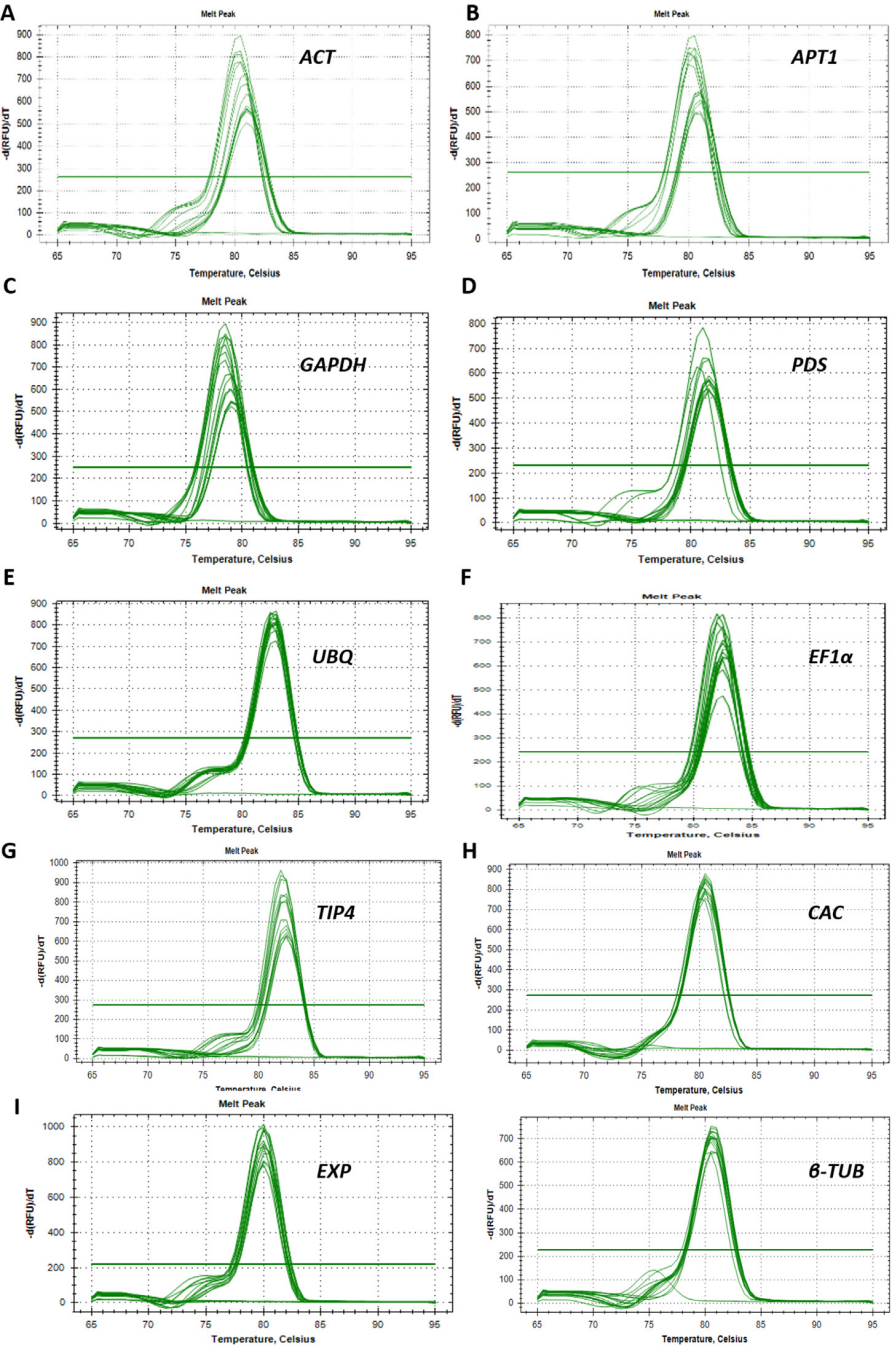
The melt curves generated and analysed for all ten-reference genes, for both susceptible and resistant tomato lines, using the CFX Manager 3.1 software. The melt curves are generated by plotting –d(RFU)/dT vs. Temperature (°C).

**Fig. 2:**
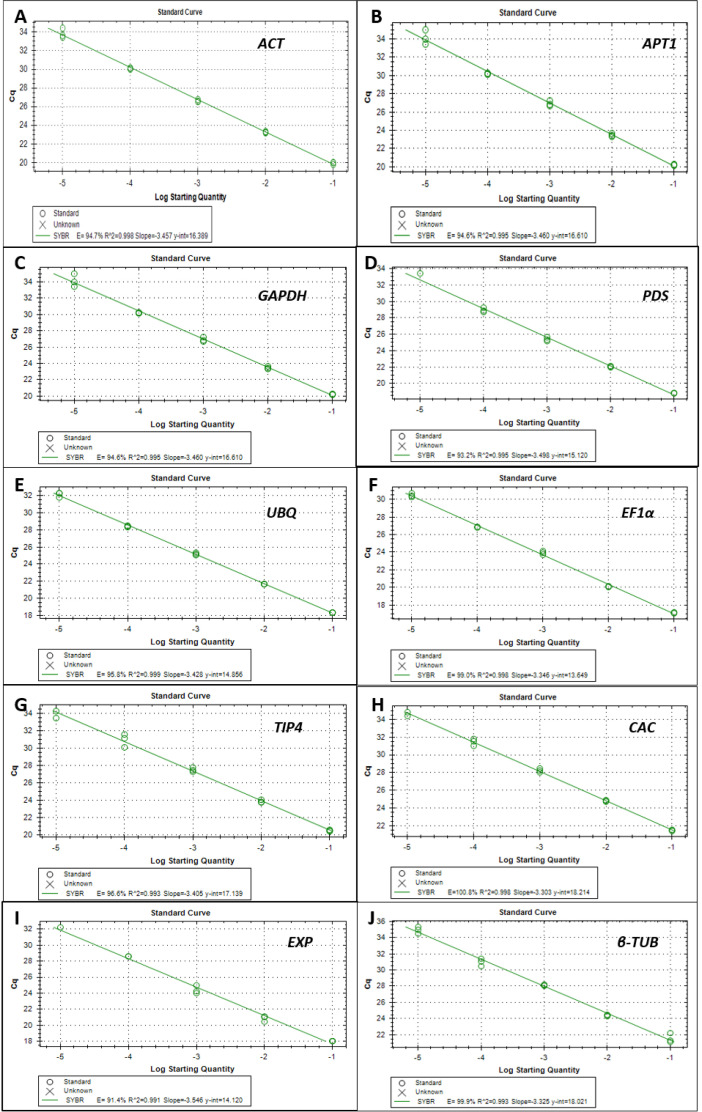
Standard curves showing the PCR efficiency values for the ten candidate reference genes, generated from a 10-fold dilution series of cDNA. (**A**) *ACT* with an E=94.7% and R^2^= 0.998 **(B)***AP1* with an E= 94.6% and R^2^ = 0.995 **(C)***GAPDH* with an E= 94.6% and R^2^ =0.995 **(D)***PDS* with an E = 93.2% and R^2^ =0.995 **(E)***UBI* with an E = 95.8% and and R^2^= 0.999 **(F)***EF1α* with an E = 99.0% and R^2^ = 0.998 **(G)***TIP4* with an E= 96.6% and R^2^ = 0.993 **(H)***CAC* with an E = 100.8% and R^2^= .0998 **(I)***EXP* with an E=91.4% and and R^2^= 0.991 **(J)***β-Tub* with an E=99.9% and R^2^=0.993.

Ten genes *ACT, EF1α, EXP, CAC, TUB, PDS, APT1, TIP41, GAPDH, and UBI* (Table 1), were selected as candidate reference genes for this experiment. The specificity of each primer pair was determined by a single peak in the melting curve analysis (Fig. 1) and further confirmed by a single band for each RT-qPCR amplicon on 2% agarose gel electrophoresis (Supplementary Fig S1). Standard curve data was generated for each primer and the PCR efficiency for each of these primer pairs was calculated using the Bio-Rad CFX manager 3.1 software (Fig 2). All candidate reference genes had an amplification efficiency of 91.4% and higher and a correlation coefficient above 0.991.(Table 1)

**Table 1:** Primers sequences for reference and target genes used for qPCR in this experiment.

Gene name	Primer sequence 5’- 3’	Amplicon size (bp)	Melting temperature (°C)	Accession number^a^
Actin 7 *(ACT)*	FWD::GGTATCCACGAGACTACCTACA REV:TGCTCATACGGTCAGCAATAC	127	81.00	Solyc11g005330.2
β-6Tubulin *(TUB)*	FWD::GCTACCTGTGGAAGGTTTGT REV:GGACGGAAGATCTGTCCATAAG	101	80.50	Solyc10g086760.2
Ubiquitin 3 *(UBI)*	FWD::CTTCGTAAGGAGTGCCCTAATG REV:GCCTCCAGCCTTGTTGTAA	117	83.00	Solyc01g056940.3
Clathrin adaptor complexes medium subunit *(CAC)*	FWD::CCTCCGTTGTGATGTAACTGG REV:ATTGGTGGAAAGTAACATCATCG	173	80.50	SGN-U314153 (Exposito-Rodriguez et al., 2008)
phytoene desaturase *(PDS)*	FWD::CAAGACCAGAGCTGGACAATAC REV:CAAACCTGCACCAGCAATAAC	119	81.50	Solyc03g123760.3
Expressed protein *(EXP)*	FWD::GCTAAGAACGTGGACCTAATG REV:TGGGTGTGCCTTTCTGAATG	183	80.50	SGN-U346908 (Exposito-Rodriguez et al., 2008)
Glyceraldehyde-3-phosphate dehydrogenase *(GAPDH)*	FWD::GGGTTGCTCTCCAAAGAAATG REV:CTGGCCGTGTACACTATCATAC	107	78.50	Solyc03g111010.3
Adenine phosphoribosyl transferase-like protein *(APT1)*	FWD::TCAGTGTGGTTGCAGGTATTG REV:CCCAGGTAACTTCTTGGGTTTC	110	81.00	Solyc04g077970.3
TAP42-interacting protein (*TIP41)*	FWD::ATGGAGTTTTTGAGTCTTCTGC REV:GCTGCGTTTCTGGCTTAGG	235	82.50	SGN-U584254 (Exposito-Rodriguez et al., 2008)
Elongation factor 1-alpha (*EF1α)*	FWD::GGCCAGATTGGAAACGGATA REV:CTTACCTGAACGCCTGTCAA	105	82.50	Solyc06g005060.3
Iron Superoxide dismutase *(SOD)*	FWD::GGCCTGGAATCATCAGTTCTT REV:GCTGCAGCTGCCTTAAATTC	138	79.50	Solyc06g048410.3
Glutathione-S-transferase *(GST)*	FWD::TGGGTTCTACTGCTGGTTTC REV:TTAGCCACACTGTCCCTTTG	117	83.00	Solyc07g056420.4
Heat shock protein *(HSC 70)*	FWD::CACCACTTTCTCTTGGGTTAGA REV:CCGGGTTGGTTATCAGAGTAAG	121	80.50	Solyc06g076020.3

a Accession numbers from Sol genomics network (SNG or Solyc : https://solgenomics.net/)

## 2 Experimental Design, Materials, and Methods

Seeds for two near-isogenic lines (NIL), susceptible line T395 (S) and resistant line T396 (R), obtained from Sakata Vegenetics R.S.A (Pty) Ltd. were grown in controlled growth chambers at 28°C with a 16-hour light and 8-hour dark periods until seedlings were 24 days old. Twenty-four day old S and R tomato seedlings were agroinocluated with infectious clones of ToCSV. Control seedlings were mock-inocluated with *Agrobacterium* harbouring an empty pCambia2300 plasmid. Four tomato plants were indepndently agroinoculated, along the stem, for each line, and the experiment was independently repeated three times.

Total RNA was extracted from infected and mock inoculated S and R leaf tissue, at 35 days post infection (dpi), using the quick RNA miniprep kit (Zymo Research, USA) as per manufacturers instruction. Total RNA was treated with DNAse (ThermoScientific, USA) and then converted to cDNA using random hexamers and oligo (dT)_18_ primers contained in Maxima H Minus First Strand cDNA Synthesis kit RT-qPCR (Thermo scientific, USA) as recommended by the manufacturer. One-in-ten (1:10) serial dilutions were prepared for the cDNA using nuclease-free PCR water before downstream qPCR runs and analysis.

Each primer pair (Table 1) were tested on cDNA synthesised for S and R using the LUNA Universal qPCR mater mix (New England Biolabs, Massachusetts). qPCR reactions were carried out in low-profile, white PCR tubes, on the CFX Connect Real Time System (Bio-Rad). For each reaction, i.e. each dilution for each gene, the qPCR was set up for four biological replicates and three technical replicates. No template water controls (NTC) were included in each qPCR run to account for any contamination. The RT-qPCR reaction was performed in a final 10 uL volume and included: 2 μL of diluted cDNA, 5 μL of 2X Luna Universal qPCR Master Mix (New-England Biolabs, Massachusetts) and qPCR gene-specific primers (Table 1) to a final concentration of 500 nM. The following amplification conditions were used: initial denaturation and enzyme activation at 95°C for 60 s, followed by 40 cycles at 95°C for 15 s, 60° C for 30 s. For melting curve analysis, a dissociation step cycle was performed at 65°C for 0.5 s followed by a gradual increase of 0.05°C for 0.5 s until 95°C. Melt curve analysis and standard curves were generated for each of the reference genes tested using the CFX Manager 3.1 software. Standard curves were generated for each gene-specific primer set and the PCR efficiency of each primer pair was calculated using the CFX manager software 3.1.

## Declaration of Competing Interest

The authors declare that they have no known competing financial interests or personal relationships that have, or could be perceived to have, influenced the work reported in this article.

## Appendix A Supplementary materials

Supplementary Data S1 Supplementary Raw Research Data. This is open data under the CC BY license http://creativecommons.org/licenses/by/4.0/ Supplementary Data S1

Supplementary Data S1 Supplementary Raw Research Data. This is open data under the CC BY license http://creativecommons.org/licenses/by/4.0/ Supplementary Data S1
