# Supplementary material for: Real-time PCR data for reference candidate gene selection in tomato infected with Tomato curly stunt virus
Source: Data Brief. 2020 May 21;31:105750. doi: 10.1016/j.dib.2020.105750 (PMC7264493; doi:10.1016/j.dib.2020.105750)
Supplement: Supplementary Data S1 — Supplementary Raw Research Data. This is open data under the CC BY license http://creativecommons.org/licenses/by/4.0/ [file mmc2.pdf]

Supplementary Data

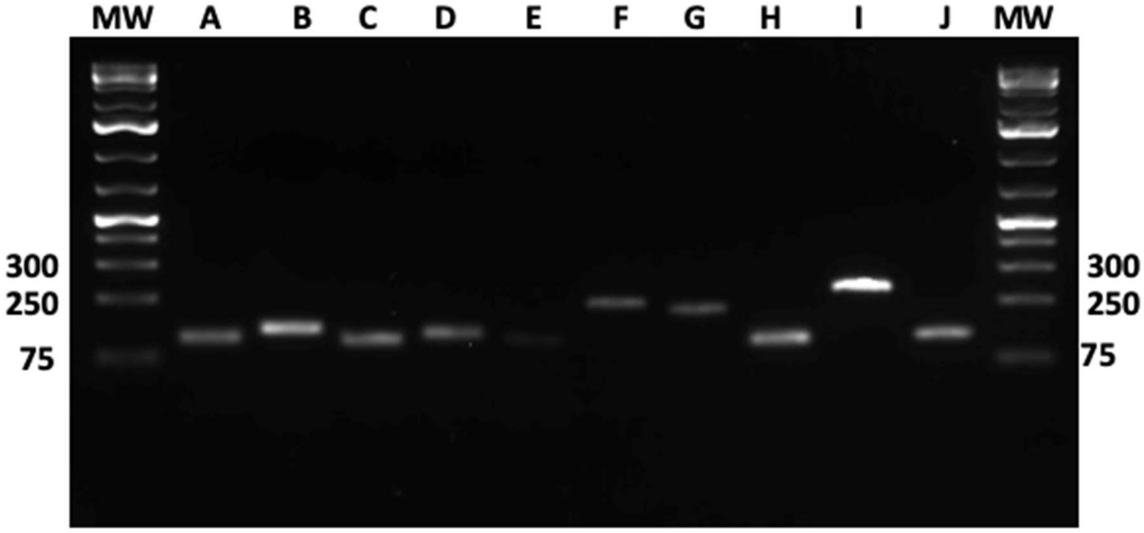

Figure S1: Agarose gel electrophoresis of showing the positive amplification of the ten candidate reference genes selected for this study (M) DNA Molecular weight Marker (1kb Plus O'GeneRuler, ThermoScientific) (A) *APT1*, (B) *ACT*, (C) *EF1α*, (D) *UBI*, (E) *TUB*, (F) *EXP*, (G) *CAC*, (H) *GAPDH*, (I) *TIP41*, (J) *PDS*. Sizes for each qPCR product is indicate in Table 1.
